# Dispersal Polymorphisms in Invasive Fire Ants

**DOI:** 10.1371/journal.pone.0153955

**Published:** 2016-04-15

**Authors:** Jackson A. Helms, Aaron Godfrey

**Affiliations:** 1 Department of Biology, University of Oklahoma, Norman, Oklahoma, United States of America; 2 ZIN Technologies, Inc., Middleburg Heights, Ohio, United States of America; University of North Carolina, Greensboro, UNITED STATES

## Abstract

In the Found or Fly (FoF) hypothesis ant queens experience reproduction-dispersal tradeoffs such that queens with heavier abdomens are better at founding colonies but are worse flyers. We tested predictions of FoF in two globally invasive fire ants, *Solenopsis geminata* (Fabricius, 1804) and *S*. *invicta* (Buren, 1972). Colonies of these species may produce two different monogyne queen types—claustral queens with heavy abdomens that found colonies independently, and parasitic queens with small abdomens that enter conspecific nests. Claustral and parasitic queens were similarly sized, but the abdomens of claustral queens weighed twice as much as those of their parasitic counterparts. Their heavier abdomens adversely impacted morphological predictors of flight ability, resulting in 32–38% lower flight muscle ratios, 55–63% higher wing loading, and 32–33% higher abdomen drag. In lab experiments maximum flight durations in claustral *S*. *invicta* queens decreased by about 18 minutes for every milligram of abdomen mass. Combining our results into a simple fitness tradeoff model, we calculated that an average parasitic *S*. *invicta* queen could produce only 1/3 as many worker offspring as a claustral queen, but could fly 4 times as long and have a 17- to 36-fold larger potential colonization area. Investigations of dispersal polymorphisms and their associated tradeoffs promises to shed light on range expansions in invasive species, the evolution of alternative reproductive strategies, and the selective forces driving the recurrent evolution of parasitism in ants.

## Introduction

Life history tradeoffs between dispersal and reproductive or competitive ability are known for many organisms [1–2], including insects [3–5]. Here we examine one such example in ant queens, in which the Found or Fly hypothesis (FoF) posits a tradeoff between colony founding and flight ability mediated by abdominal nutrient loads [6]. In most species young queens fly from their natal nests to mate and disperse [7–8]. After finding a suitable nest site a queen sheds her wings, lays eggs and grows a new colony [9]. Heavier abdomens, containing more fat and protein reserves [10–12], increase a founding queen’s survival and reproductive output [13–15]. At the same time, heavier nutrient loads negatively impact flight morphology by decreasing flight muscle ratio (FMR) and increasing wing loading and abdomen drag [6], changes which likely hinder a queen’s ability to disperse long distances or search for nest sites [16–17].

This tradeoff is both ecological, playing out during an individual queen’s lifetime as she gains abdominal weight [6], and evolutionary, causing species with different reproductive strategies to differ also in flight morphology [18]. The evolution of the most common strategy, claustral founding, in which founding queens are isolated and survive entirely off their abdominal reserves [11, 19–20], likely incurs a cost in dispersal ability [18]. At the opposite extreme, socially parasitic species found colonies inside the nests of other ants and manipulate the native workers into raising foreign offspring [21–22]. Because parasitic queens enter fully functioning colonies, complete with food reserves and foraging workers, they store no abdominal nutrients and are unable to found colonies independently [11] but may be better dispersers. Parasitism is common among ants, with over 200 known parasitic species arising from dozens of independent origins [21–22], and parasites may constitute up to a third of the ant species in some regions [21]. Many species are facultatively parasitic, capable of producing two different queen types within the same colony and from the same genome [23–26]. In these queen polymorphic species, one queen type founds colonies in the typical claustral manner and the other founds colonies parasitically by entering conspecific nests. The two queen types often fly and mate at different times of the year and may also differ in size or morphology [21–22, 25]. In the simplest cases the queen types differ only in abdomen weight [27]. Queen polymorphic ant species are thus ideal systems for studying reproduction-dispersal tradeoffs, as they allow us to isolate the effects of reproductive strategy and nutrient loading while controlling for evolutionary history, ecology, geography and even genetic variation.

**Fig 1:**
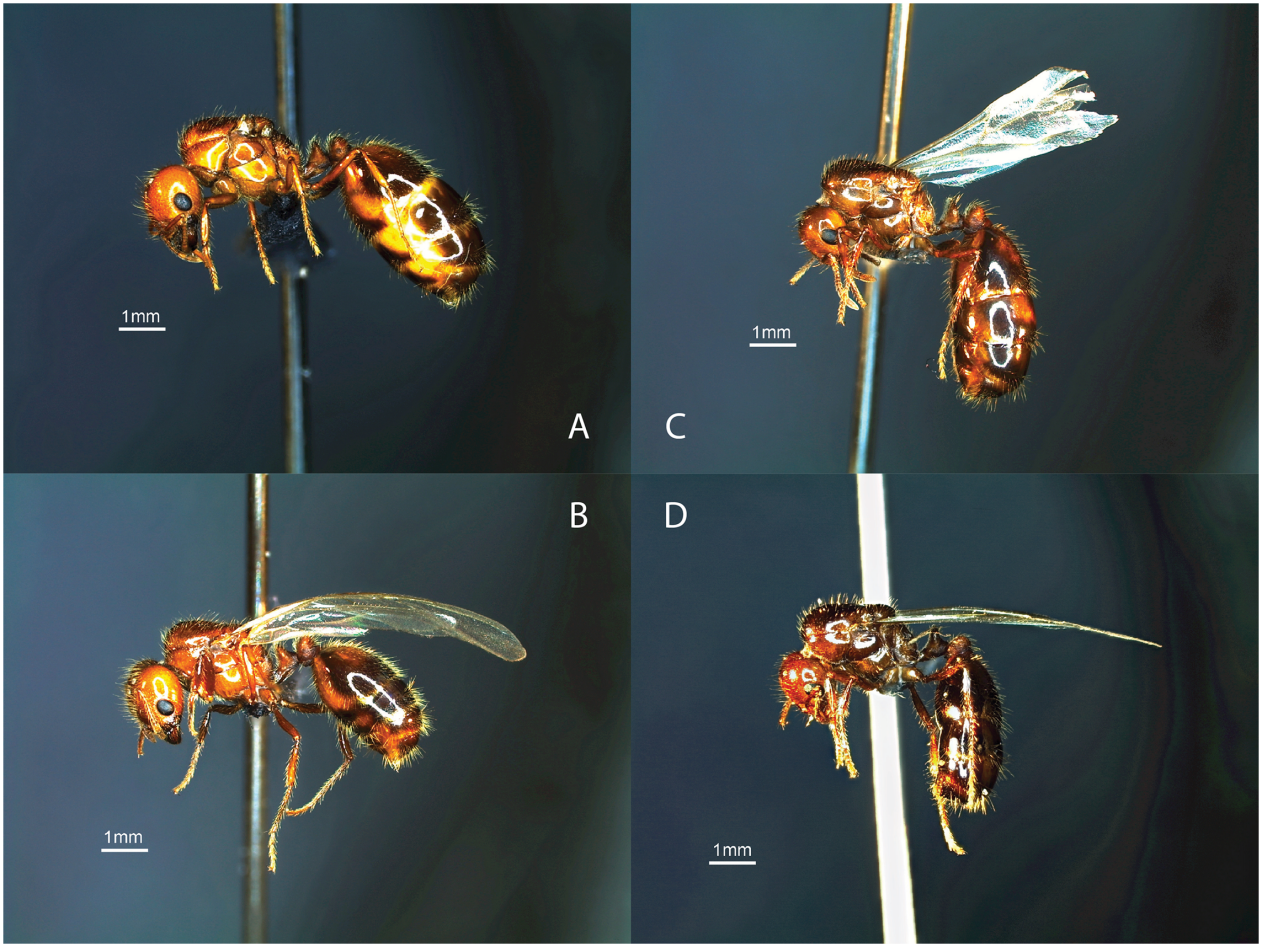
Queens of *Solenopsis geminata* (A, B) and *S*. *invicta* (C, D). In both species claustral queens (A, C) store more nutrients and have larger abdomens than parasitic queens (B, D) (Photos by Brittany Benson).

Differences in size, morphology, or mating season among polymorphic queens may be associated with differences in dispersal ability [28–31]. Here we examine two queen polymorphic fire ant species that co-occur [32] in the southeastern United States—the tropical fire ant (*Solenopsis geminata* Fabricius, 1804) and the red imported fire ant (*S*. *invicta* Buren, 1972) (Fig 1). Dispersal studies are particularly relevant in these cases because both fire ants are global invaders whose non-native ranges are currently expanding through active dispersal during mating flights [33–35]. The two species are facultative parasites, with colonies producing both claustral and parasitic queens that differ primarily in abdomen weight and fly at different times of the year [27, 36]. Colonies produce claustral queens during the spring and summer to found new colonies independently. The lighter parasitic queens, in contrast, fly and mate in the fall (*S*. *geminata*) or late winter (*S*. *invicta*), enter conspecific colonies whose queens happen to have died during the year, and manipulate the orphaned workers into adopting them as their new queen.

The two species differ, however, in how queens become claustral or parasitic. In *S*. *geminata* the two queen types experience slightly different developmental trajectories [36], allowing their morphologies to diverge in response to dispersal tradeoffs. For example, claustral queen types might compensate for their heavier abdomens, as claustral species do in interspecific comparisons, by developing larger wings than parasitic queens [18]. In *S*. *invicta*, in contrast, queen type appears not to be developmentally determined, as the queens differ only in adult weight gain and behavior [27, 37]. Queen types in *S*. *invicta* therefore represent alternate ways to use the same underlying body, precluding evolutionary alterations to one queen type’s morphology independently of the other. The similarities between the species thus provide two replicate study systems, while their differences allow us to explore the dispersal consequences of different modes of caste determination.

We test for dispersal polymorphisms in these species in the context of the Found or Fly hypothesis using a three-pronged approach—we first test our assumptions, then we test for flight morphology differences among queen types, and finally we link flight morphology to flight performance. FoF assumes A1) that queen types differ in abdomen mass due to the different energetic loads required for reproduction, and A2) that heavier abdomens adversely impact flight morphology. Because of the abdomen mass differences, FoF predicts that claustral queens will have P1) lower flight muscle ratios, P2) higher wing loading, and P3) higher abdomen drag. Insect wings are evolutionarily labile [38], however, and populations may break the wing loading tradeoff by evolving larger wings [18]. We therefore predict that in *S*. *geminata*, in which queen types differ developmentally, P4) claustral queens will develop larger wings to compensate for higher abdomen loads. In contrast, in *S*. *invicta*, in which queen types experience the same developmental program, P5) queen morphs will not differ in wing size. Translating these morphological differences into dispersal ability, we predict queens with heavier abdomens will P6) have shorter flight durations. We test these predictions by studying the morphology and flight behavior of naturally varying queens. Finally, we combine our results in a simple model that links reproductive strategy, abdomen mass, reproductive output, and flight ability.

## Materials and Methods

### Locality and specimens

All specimens were collected and all experiments performed in 2013 in and around Tallahassee, northern Florida, USA (30°27’18”N 84°15’12”W). Both target fire ant species co-occur here [32], *S*. *geminata* as a native or ancient invasive species [39] and *S*. *invicta* as a recent invasive [34]. Studied populations of both species were monogyne (having one queen per colony), although polygyne *S*. *geminata* have been collected in Florida [39–40] and polygyne *S*. *invicta* occur in the Tallahassee area at low frequencies [41]. No permits were required to sample the ants, as they were hand collected along public roadsides, and no protected or endangered species were involved.

### Flight morphology

To test morphological assumptions and predictions we collected virgin queens that had not yet flown, either from on top of their nests as they left for their mating flights or by excavating them from the upper layers of mature nests during the mating season, and preserved them in ethanol. We collected parasitic *S*. *invicta* in late winter (13 to 17 March), claustral *S*. *invicta* and claustral *S*. *geminata* in late spring and summer (18 to 25 June and 18 June to 12 July), and parasitic *S*. *geminata* in autumn (20 to 21 November). We collected *S*. *geminata* queens from sandy soils in longleaf pine (*Pinus palustris*) savannas within Apalachicola National Forest, and *S*. *invicta* queens from lawns and roadsides in the Tallahassee area. For each queen type we sampled 13 to 58 individuals representing three to six separate colonies and measured their flight morphology. Differences among queen castes in these species are comparable to those among heterospecific queens, which can be detected with sample sizes as low as three to six [18]. Pilot studies showed that variance in fire ant flight morphology measurements stabilized after about 7 individuals (mean 6.9 ±4.7, *n* = 16 morphology by queen type measurements).

We processed each specimen according to a protocol adapted from [6]. For the sake of clarity we refer to the mesosoma as the *thorax* and the gaster as the *abdomen*. To measure body and abdomen size we measured head width, abdomen length and abdomen height to 0.1 mm with an ocular micrometer under a dissecting microscope. Head width—the maximum width of the head in full-face view excluding the eyes—is a standard measure of ant body size. Abdomen length is the maximum length of the abdomen measured from the dorsal point of attachment of the post-petiole. Abdomen height is the maximum height of the abdomen in profile view. After linear measurements we separated the abdomen, thorax, wings and other body parts and dried them for 48 to 72 hrs at 60 to 65°C. We weighed the dried abdomen, thorax, wings and entire body to 0.001 mg using a Cahn microbalance. After weighing we placed one forewing and one hindwing from each specimen onto a slide and photographed them with a reference ruler under a Leica dissecting microscope camera. We then measured wing lengths and wing areas using ImageJ software [42].

After processing the specimens we calculated flight morphology metrics for each queen. Flight muscle ratio (FMR)—the ratio of flight muscle mass to body mass—is probably the most important predictor of insect flight performance [43–46]. FMR is proportional to acceleration and load lifting ability, and a higher FMR increases maneuverability, flight endurance and the temperature range at which an insect can fly. We calculated FMR by dividing the thorax mass by total body mass. Thorax mass is a standard surrogate for flight muscle in ants and other insects [47–49]. To ensure queen types did not differ in flight muscle development we dissected a voucher specimen of each type to look for atrophied or absent flight muscle. In all cases flight muscle was well developed and filled the thorax, justifying the use of thorax mass as a surrogate for flight muscle mass. Another metric, wing loading—the ratio of body weight to wing area—decreases maneuverability, flight endurance and maximum flight speed, and increases minimum power and speed requirements for flight [50–54]. We calculated wing loading by dividing body mass by the combined area of all four wings (mg/mm^2^). A third metric, abdomen drag, increases the power requirements of flight and reduces overall flight performance [45–46]. Drag is determined by an object’s size and shape and is proportional to a two-dimensional reference area. We use a volumetric reference area—abdomen volume^2/3^ (mm^2^)—which links mass to size and shape [55–56]. We calculated abdomen volume using the formula for a prolate spheroid, using abdomen length and height as the major and minor axes. Finally, we calculated two aspects of wing morphology that are independent of abdomen mass and hence not likely to vary with reproductive strategy. Aspect ratio—wing narrowness—equals 4*forewing length^2^/total wing area. Narrower wings—higher aspect ratios—increase aerodynamic efficiency [46, 50, 57]. Wing mass density—a measure of stiffness and durability—is total wing mass divided by total wing area (mg/mm^2^).

To control for body size differences when comparing flight morphology we first checked whether parasitic and claustral queens differed in head width. We then tested whether parasitic queens had lighter abdomens than claustral queens (A1). To examine how abdomen mass impacts flight morphology (A2) we regressed flight muscle ratio, wing loading and drag against abdomen mass separately for each queen type. We then tested flight morphology predictions (P1 to P5) by comparing queen types (Table 1).

**Table 1:** Flight morphology terms and predictions.

Trait	Definition	Predictions
Flight muscle ratio (FMR)	Ratio of flight muscle mass to body mass. Increases maneuverability, acceleration, load lifting ability, and the ability to fly at cooler temperatures.	Lower in claustral queens
Wing loading (mg/mm^2^)	Ratio of body mass to wing area. Decreases maneuverability, endurance, and maximum flight speed. Increases power and speed requirements.	Higher in claustral queens
Abdomen drag (mm^2^)	Theoretical area proportional to the drag experienced in flight. Increases power requirements and reduces performance.	Higher in claustral queens
Wing area (mm^2^)	Area of all four wings	*S*. *geminata*: Higher in claustral queens. *S*. *invicta*: No difference between types.
Forewing length (mm)	Length of the front wings	*S*. *geminata*: Higher in claustral queens. *S*. *invicta*: No difference between types.
Aspect ratio	Wing narrowness. Increases aerodynamic efficiency.	No difference between types
Wing mass density (mg/mm^2^)	Wing stiffness or durability.	No difference between types
Flight duration (s)	The amount of time tethered queens fly during six consecutive take off events.	Decreases with abdomen mass; increases with flight muscle ratio; decreases with wing loading

### Live flight

We followed up flight morphology comparisons with a live flight experiment to link morphology to dispersal ability. The experiment was performed from June to July using claustral *S*. *invicta* queens. We used claustral *S*. *invicta* queens because they were reliably available in sufficient numbers, perform well in lab conditions, and are routinely used as models in ant biology [34]. We collected virgin queens that had not yet flown by excavating them from the top layers of mature colonies in the morning, along with soil and workers from the nest. These colony fragments were kept in plastic containers in the lab and given water. To avoid weight loss or other effects of captivity on flight [58], queens participated in experiments within three days of their collection. Colony fragments remained vigorous and displayed normal behavior throughout this time. Flight experiments were performed in indoor chambers linked to the outside environment through screened windows. Temperature, humidity and barometric pressure thus reflected normal mating season weather conditions but with strong air currents eliminated.

To examine how abdomen mass impacts flight endurance (P6) we observed 33 queens from three colonies during tethered flight [59–61]. Flights were performed from 0900 to 1800 at temperatures ranging from 27.0 to 29.6°C and relative humidity ranging from 67 to 79%, approximating the natural range of flight conditions [34]. We tied a 30.5 cm lightweight (0.117 mg/cm) polyester string around the petiole of each queen. About 2.5 cm were used in tying, leaving a 28 cm tether. We clipped the tether to the end of a wooden rod projecting 25 cm horizontally from a table top 75 cm above the ground. We induced queens to fly by gently scraping them off a wooden stick or by blowing on them. Once a queen took off we timed her with a stop watch until she either landed on the rod or stopped flying and hung from the tether. We made each queen fly for six consecutive trials or until she would not take off, and filmed flights with a digital camcorder. For each queen we added all flight durations together to calculate a total flight time. We used total flight time because we are interested in a queen’s maximum dispersal performance, but total flight time and average time per bout are tightly correlated (*r*^*2*^ = 0.98) and the results would be similar for either measure. After the flights we preserved queens in ethanol and processed them as above to compare their performance to their flight morphology. We removed as an outlier one queen with an anomalously light abdomen who had likely just eclosed and was not prepared for her mating flight, leaving 32 queens for analysis.

Factors other than biomechanical considerations likely influence flight duration, such that queens with high potential flight endurance may still fly for only a short time. We thus predicted that queens with heavy abdomens, and thus lower flight muscle ratios and higher wing loading, could have only short flights but those with light abdomens could have long or short flights. In other words, the maximum and range of total flight times should decrease in queens with heavier abdomens (Table 1). Quantile regressions are ideal for characterizing such heterogeneous relationships [62]. In our case, we used quantile regressions through the upper quartile to compare maximum values of total flight time to abdomen mass, flight muscle ratio and wing loading. Maximum performance is particularly relevant in ant dispersal studies, as long distance dispersal events impact colony founding success and population occurrence [63], especially during the spread of invasives in novel environments [34]. Maximum dispersal ability is of additional applied importance in the case of *S*. *invicta*, as its rate of spread in the United States (up to 48 km/yr [64]) has exceeded by an order of magnitude estimates of its dispersal ability (<1.6 to 5.4 km [52, 65]), due to long distance dispersal events [34, 66]. Despite our primary interest in maximum flight performance, however, we also performed ordinary least squares regressions and nonparametric Spearman’s rank correlations as measures of central tendency and for comparison to the upper quartile results.

As an additional measure of flight performance we attempted to measure queen flight distances when dropped. Fifty-six queens were dropped from a height of 170 cm above the center of a 2 x 2 meter chamber, and their resultant flight distances measured. This experiment detected a possible unimodal relationship between abdomen mass and flight distance. The explanatory power, however, was low (*r*^*2*^ = 0.13), likely because of the unrealistic limits of the flight chamber and method of flight initiation (dropping from a height versus taking off from a surface), and we excluded it from our results as uninformative.

### Tradeoff model

To further explore the tradeoff between reproduction and dispersal we translated our live flight results to the complete range of parasitic and claustral *S*. *invicta* abdomen masses by extending the curve derived from our tethered flight experiment. As a measure of reproductive output over the same range we adapted a formula that relates claustral *S*. *invicta* abdomen mass to the production of first generation workers [67–68]. Early workers are reared entirely from queen nutrient reserves, and the relationship was determined by planting incipient colonies headed by queens of different weights and observing their reproductive output [68]. For flight speed comparisons we used a formula that relates tethered *S*. *invicta* flight speeds to total body mass [52]. In constructing this speculative model we make several assumptions. First, in extending the flight endurance curve we assume that flight time, rather than dropping to zero, levels off at about 160 seconds in the heaviest queens, a realistic flight time as *S*. *invicta* queens often fly less than 400 meters [34]. Second, to adapt the worker production curve, which applies to live weight instead of dry weight, we assume a live to dry weight ratio of two [69]. Third, we assume that abdomen mass increases consist of fat and protein that is all converted to offspring production. Finally, we assume that patterns derived from claustral queens apply equally to parasitic queens of the same species.

### Data analysis

Throughout our analyses we treat individual queens as independent samples. Because we usually measured multiple queens per colony, however, many individuals were sisters whose morphology or flight performance may not have been independent from that of other queens. We dealt with this in several ways. In the case of flight morphology comparisons, we repeated all our analyses using colony averages rather than individual queens, treating each sampled colony as a single data point. When analyzing flight durations, we included colony identity as a factor in all regressions but it was never significant. We also tested whether colonies used in the flight experiments differed in any of the variables analyzed, and found that they did not. We therefore excluded colony identity as a factor in the final flight duration analyses

All statistics were performed in R [70]. We checked normality of variables with the Shapiro-Wilk test. Paired comparisons used *t*-tests for normally distributed variables, presented as means and standard deviations, and Kruskal-Wallis tests for non-normal variables, presented as medians and interquartile ranges (IQR). For the tethered flight experiment, flight muscle ratio was log transformed to meet normality assumptions. Quantile regressions were performed with the *quantreg* R package [71]. To account for experimentwise error we applied the Holm-Bonferroni correction [72] to *p*-values of regressions of flight morphology versus abdomen mass within queen types, and to regressions of flight duration versus morphology.

## Results

### Flight morphology

**Fig 2:**
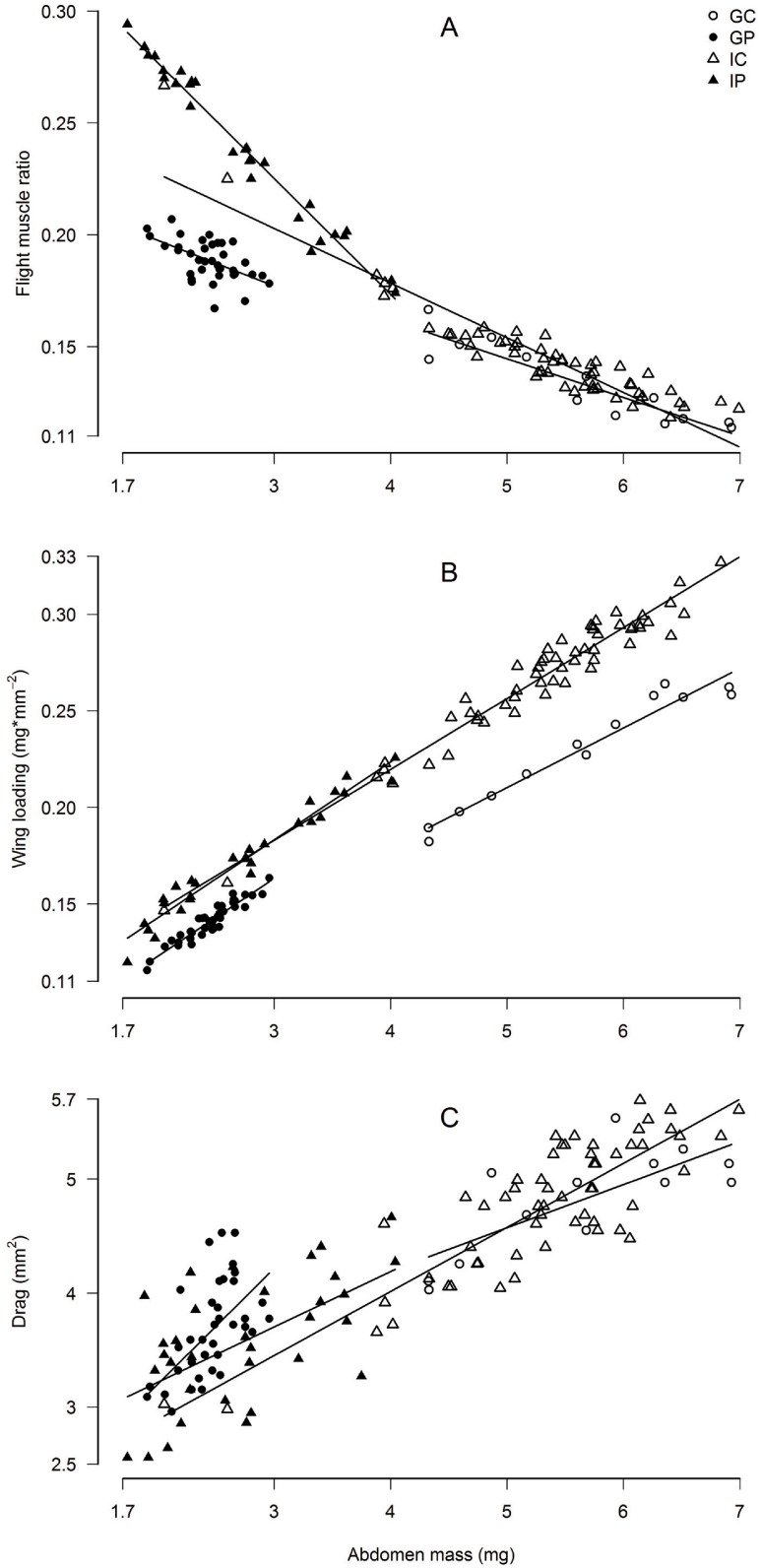
Queen flight morphology and abdomen mass. For all queen morphs heavier abdomens impact flight morphology by (A) decreasing flight muscle ratio, (B) increasing wing loading, and (C) increasing abdomen drag. GC = *S*. *geminata* claustral, GP = *S*. *geminata* parasitic, IC = *S*. *invicta* claustral, IP = *S*. *invicta* parasitic.

We compared the flight morphology of 142 queens from three mating seasons throughout the year (13 claustral *S*. *geminata*, 38 parasitic *S*. *geminata*, 58 claustral *S*. *invicta*, and 33 parasitic *S*. *invicta*, Table 2A). Head widths did not differ between queen types in *S*. *geminata* (Kruskal-Wallis *p* = 0.062, parasite median 1.6 mm, IQR = 1.5–1.6, claustral median 1.6 mm, IQR = 1.6–1.6) or *S*. *invicta* (Kruskal-Wallis *p* = 0.078, parasite median 1.4 mm, IQR 1.35–1.40, claustral median 1.4 mm, IQR 1.4–1.5). Despite having similar body sizes, the abdomens of claustral *S*. *geminata* queens (A1) were 2.3 times heavier than those of parasitic queens (5.652 ±0.93 mg versus 2.453 ±0.24 mg, *p* = 2.3 x 10^−8^). Likewise, abdomens of claustral *S*. *invicta* averaged nearly double the weight of their parasitic counterparts (5.331 ±0.91 mg versus 2.745 ±0.67, *p* = 2.2 x 10^−16^). We obtained similar results when comparing averages among colonies (Table 2B). Colony average head widths did not differ between queen types in *S*. *geminata* (*p* = 0.77, parasite mean 1.59 ±0.090 mm, claustral mean 1.58 ±0.071 mm) or *S*. *invicta* (*p* = 0.12, parasite mean 1.37 ±0.063 mm, claustral mean 1.44 ±0.054 mm), and abdomens of claustral queens were 2.5 times heavier than parasitic queens in *S*. *geminata* (*p* = 0.002, claustral mean 5.856 ±0.46 mg, parasitic mean 2.342 ±0.24 mg) and 2.1 times greater in *S*. *invicta* (*p* = 7.9 x 10^−5^, claustral mean 5.555 ±0.50 mg, parasitic mean 2.672 ±0.74 mg). Within each queen type heavier abdomens adversely impacted flight morphology (A2) by decreasing flight muscle ratio by 11 to 53%, increasing wing loading by 35 to 122%, and increasing drag by 23 to 95% over their respective ranges of abdomen mass (Fig 2 and Table 3).

**Table 2:** Fire ant queen flight morphology.

A. Individual averages				
	*Solenopsis geminata*		*Solenopsis invicta*	
	Claustral	Parasitic	Claustral	Parasitic
*n*	13	38	58	33
Head width (mm)	1.60 (0.058)	1.56 (0.076)	1.41 (0.075)	1.39 (0.062)
Dry mass (mg)	7.751 (0.95)	4.101 (0.35)	7.242 (0.95)	4.690 (0.75)
Abdomen mass (mg)	5.652 (0.93)	2.453 (0.24)	5.331 (0.91)	2.745 (0.67)
Flight muscle ratio	0.13 (0.018)	0.19 (0.009)	0.15 (0.024)	0.24 (0.035)
Wing loading (mg/mm2)	0.230 (0.029)	0.141 (0.010)	0.268 (0.034)	0.173 (0.028)
Forewing length (mm)	7.19 (0.14)	6.66 (0.10)	6.48 (0.10)	6.57 (0.12)
Total wing area (mm2)	33.7 (1.1)	29.1 (0.9)	27.0 (0.9)	27.6 (1.0)
Aspect ratio	6.14 (0.19)	6.11 (0.10)	6.23 (0.15)	6.24 (0.17)
Wing mass density (mg/mm2)	0.0042 (0.002)	0.0050 (0.0006)	0.0047 (0.001)	0.0059 (0.001)
Abdomen drag (mm2)	4.82 (0.46)	3.66 (0.42)	4.76 (0.60)	3.58 (0.56)
B. Colony averages				
	*Solenopsis geminata*		*Solenopsis invicta*	
	Claustral	Parasitic	Claustral	Parasitic
*n*	3	5	4	6
Head width (mm)	1.58 (0.071)	1.59 (0.090)	1.44 (0.054)	1.37 (0.063)
Dry mass (mg)	7.957 (0.51)	4.022 (0.36)	7.504 (0.55)	4.642 (0.80)
Abdomen mass (mg)	5.856 (0.46)	2.342 (0.24)	5.555 (0.50)	2.672 (0.74)
Flight muscle ratio	0.13 (0.005)	0.19 (0.006)	0.14 (0.0008)	0.24 (0.039)
Wing loading (mg/mm2)	0.239 (0.018)	0.136 (0.010)	0.274 (0.013)	0.168 (0.034)
Forewing length (mm)	7.12 (0.14)	6.69 (0.11)	6.49 (0.057)	6.58 (0.082)
Total wing area (mm2)	33.4 (1.0)	29.5 (1.2)	27.3 (0.77)	27.9 (1.1)
Aspect ratio	6.08 (0.17)	6.08 (0.055)	6.19 (0.11)	6.20 (0.16)
Wing mass density (mg/mm2)	0.0043 (0.001)	0.0051 (0.0002)	0.0050 (0.001)	0.0058 (0.001)
Abdomen drag (mm2)	4.93 (0.30)	3.42 (0.37)	4.91 (0.35)	3.44 (0.65)

Values are means, parentheses show standard deviations.

**Table 3:** Ordinary least squares regressions of queen flight morphology on abdomen mass.

	Queen	*n*	Slope	Intercept	*r2*	*p*	Corr. *p*
FMR	GC	13	-0.0173	0.231	0.8467	8.4 x 10^−6^	1.7 x 10^−5^
	GP	38	-0.0205	0.238	0.3151	2.5 x 10^−4^	2.5 x 10^−4^
	IC	58	-0.0244	0.276	0.857	2.0 x 10^−16^	8.0 x 10^−16^
	IP	28	-0.0518	0.380	0.9717	2.0 x 10^−16^	8.0 x 10^−16^
Wing loading	GC	13	0.0309	0.0558	0.9523	1.3 x 10^−8^	1.3 x 10^−8^
	GP	38	0.0403	0.0423	0.8981	2.0 x 10^−16^	8.0 x 10^−16^
	IC	56	0.0367	0.0727	0.9482	2.0 x 10^−16^	8.0 x 10^−16^
	IP	28	0.0403	0.0621	0.9538	2.0 x 10^−16^	8.0 x 10^−16^
Drag	GC	13	0.379	2.678	0.5772	0.003	2.6 x 10^−3^
	GP	38	1.015	1.171	0.3425	0.0001	3.4 x 10^−4^
	IC	58	0.562	1.767	0.7258	2.0 x 10^−16^	8.0 x 10^−16^
	IP	33	0.485	2.247	0.3425	0.0003	6.9 x 10^−4^

*GC* = claustral *S*. *geminata*, *GP* = parasitic *S*. *geminata*, *IC* = claustral *S*. *invicta*, *IP* = parasitic *S*. *invicta*

*Corr*. *p* shows *p* values corrected for experimentwise error.

**Fig 3:**
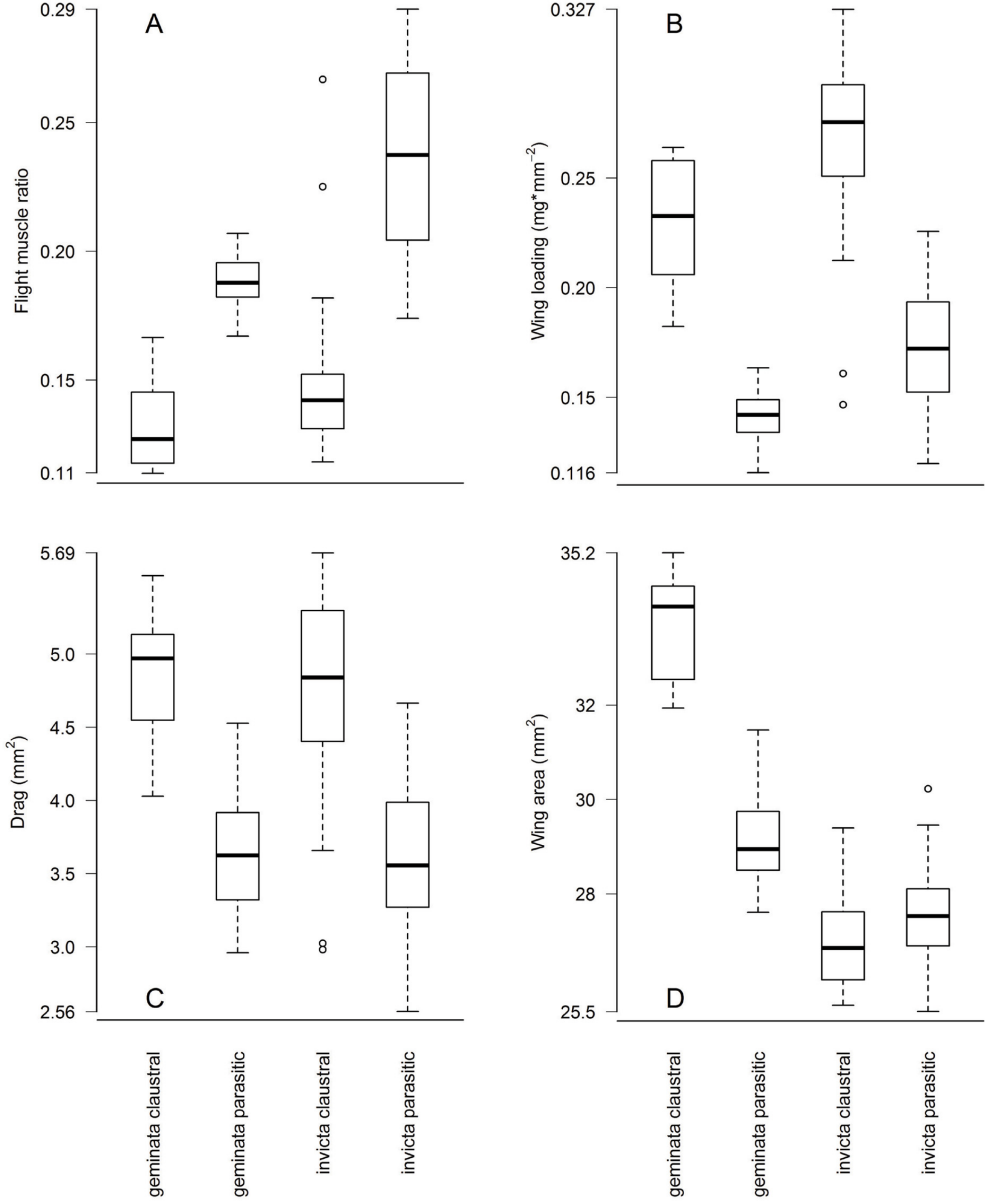
Claustral versus parasitic flight morphology (individual queens). Heavier abdomens mean claustral queens experience (A) lower flight muscle ratios, (B) higher wing loading, and (C) higher abdomen drag than parasitic queens of the same species. (D) Claustral founders in *S*. *geminata* have evolved larger wings than parasitic queens, compensating somewhat for the wing loading effects of heavier abdomens. In *S*. *invicta*, however, there is either no difference in wing size (see text) or claustral queens have slightly smaller wings.

**Fig 4:**
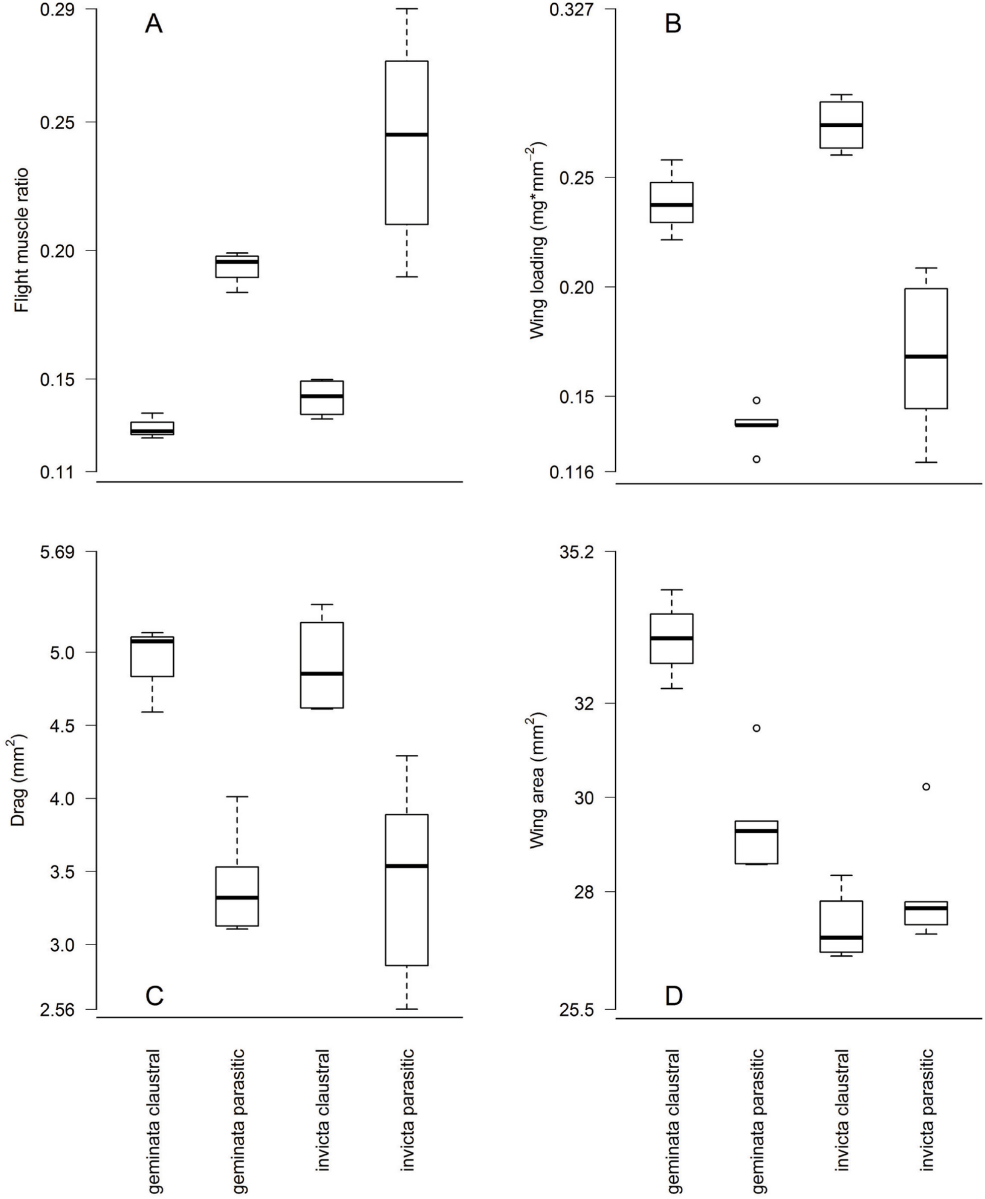
Claustral versus parasitic morphology (colony averages). Similar results apply when comparing colony averages. Claustral queens have (A) lower flight muscle ratios, (B) higher wing loading, and (C) higher abdomen drag than parasitic queens of the same species. (D) Claustral founders in *S*. *geminata* have evolved larger wings than parasitic queens, but in *S*. *invicta* there is no difference in wing size among queen types.

Flight muscle ratios, wing loading and abdomen drag varied among queens types in the predicted directions. Due to their heavier abdomens flight muscle ratios (P1) of claustral *S*. *geminata* queens were 32% lower (0.13 ±0.018 versus 0.19 ±0.009, *p* = 2.9 x 10^−8^), and those of claustral *S*. *invicta* queens 38% lower (0.15 ±0.024 versus 0.24 ±0.035, *p* = 2.0 x 10^−15^), than their parasitic counterparts (Fig 3A). Likewise, wing loading (P2) was 63% higher in claustral *S*. *geminata* (0.230 ±0.029 versus 0.141 ±0.010 mg/mm^2^, *p* = 7.9 x 10^−8^) and 55% higher in claustral *S*. *invicta* (0.268 ±0.034 versus 0.173 ±0.028 mg/mm^2^, *p* = 2.2 x 10^−16^) than in parasitic queens of the same species (Fig 3B). Larger abdomens resulted in 32% higher drag (P3) in claustral *S*. *geminata* (4.82 ±0.46 versus 3.66 ±0.42 mm^2^, *p* = 1.7 x 10^−7^) and 33% higher drag in claustral *S*. *invicta* (4.76 ±0.60 versus 3.58 ±0.56 mm^2^, *p* = 3.3 x 10^−14^) than in parasitic queens (Fig 3C). The same results apply when comparing colony averages. Colony average flight muscle ratios were 32% lower in claustral *S*. *geminata* (0.13 ±0.005 versus 0.19 ±0.006, *p* = 1.4 x 10^−5^) and 42% lower in claustral *S*. *invicta* (0.14 ±0.0008 versus 0.24 ±0.039, *p* = 0.001) than in their parasitic counterparts (Fig 4A). Colony average wing loading was 76% higher in claustral *S*. *geminata* (0.239 ±0.018 versus 0.136 ±0.010 mg/mm^2^, *p* = 0.004) and 63% higher in claustral *S*. *invicta* (0.274 ±0.013 versus 0.168 ±0.034 mg/mm^2^, *p* = 0.0002) than in parasitic queens (Fig 4B). Finally, colony average drag was 44% higher in claustral *S*. *geminata* (4.93 ±0.30 versus 3.42 ±0.37 mm^2^, *p* = 0.001) and 43% higher in claustral *S*. *invicta* (4.91 ±0.35 versus 3.44 ±0.65 mm^2^, *p* = 0.002) than in their parasitic counterparts (Fig 4C). These flight morphology differences are robust, with large effect sizes. Nevertheless, due to the small number of colonies studied, further sampling may help refine these results.

Wing morphology also differed among queen types as predicted. Claustral *S*. *geminata* wing areas (P4) were 16% larger (33.7 ±1.1 versus 29.1 ±0.9 mm^2^, *p* = 1.1 x 10^−10^) and their forewings 8% longer (7.19 ±0.14 versus 6.66 ±0.10 mm, *p* = 6.1 x 10^−10^) than in parasitic queens (Fig 3D). Similarly, when comparing colony averages, claustral *S*. *geminata* wings were 13% larger (33.4 ±1.0 versus 29.5 ±1.2 mm^2^, *p* = 0.005) and 6% longer (7.12 ±0.14 versus 6.69 ±0.11 mm, *p* = 0.016) than in parasitic queens (Fig 4D). Claustral *S*. *invicta* queens (P5), in contrast, had slightly smaller (27.0 ±0.9 versus 27.6 ±1.0 mm^2^, *p* = 0.004) and shorter wings (6.48 ±0.10 versus 6.57 ±0.12 mm, *p* = 0.002) than parasitic queens (Fig 3D). The differences in *S*. *invicta*, however, were small—only a 2% difference in wing area (0.6 mm^2^) and 1% difference in forewing length (0.09 mm)—and disappeared when comparing colony averages (wing area: 27.3 ±0.77 versus 27.9 ±1.1 mm^2^, *p* = 0.28, Fig 4D; forewing length: 6.49 ±0.057 versus 6.58 ±0.082 mm, *p* = 0.07). Aspect ratios did not differ among queen types in either species (*S*. *geminata* claustral 6.14 ±0.19 versus parasitic 6.11 ±0.10, *p* = 0.57; *S*. *invicta* claustral 6.23 ±0.15 versus parasitic 6.24 ±0.17, *p* = 0.76) and neither did wing mass density in *S*. *geminata* (claustral 0.0042 ±0.002 versus parasitic 0.0050 ±0.0006 mg/mm^2^, *p* = 0.09). These similarities held when comparing colony averages. Colony average aspect ratios did not differ among queen types in either species (*S*. *geminata* claustral 6.08 ±0.17 versus parasitic 6.08 ±0.055, *p* = 0.97, *S*. *invicta* claustral 6.19 ±0.11 versus parasitic 6.20 ±0.16, *p* = 0.90), and neither did wing mass density in *S*. *geminata* (claustral 0.0043 ±0.001 versus parasitic 0.0051 ±0.0002 mg/mm^2^, *p* = 0.12). Individual claustral *S*. *invicta* queens, on the other hand, had lighter wings than parasitic queens (0.0047 ±0.001 versus 0.0059 ±0.001 mg/mm^2^, *p* = 3.5 x 10^−5^), but again the difference is slight (0.0012 mg/mm^2^) and disappeared when comparing colony averages (claustral 0.0050 ±0.001 versus parasitic 0.0058 ±0.001, *p* = 0.06).

### Live flight

**Fig 5:**
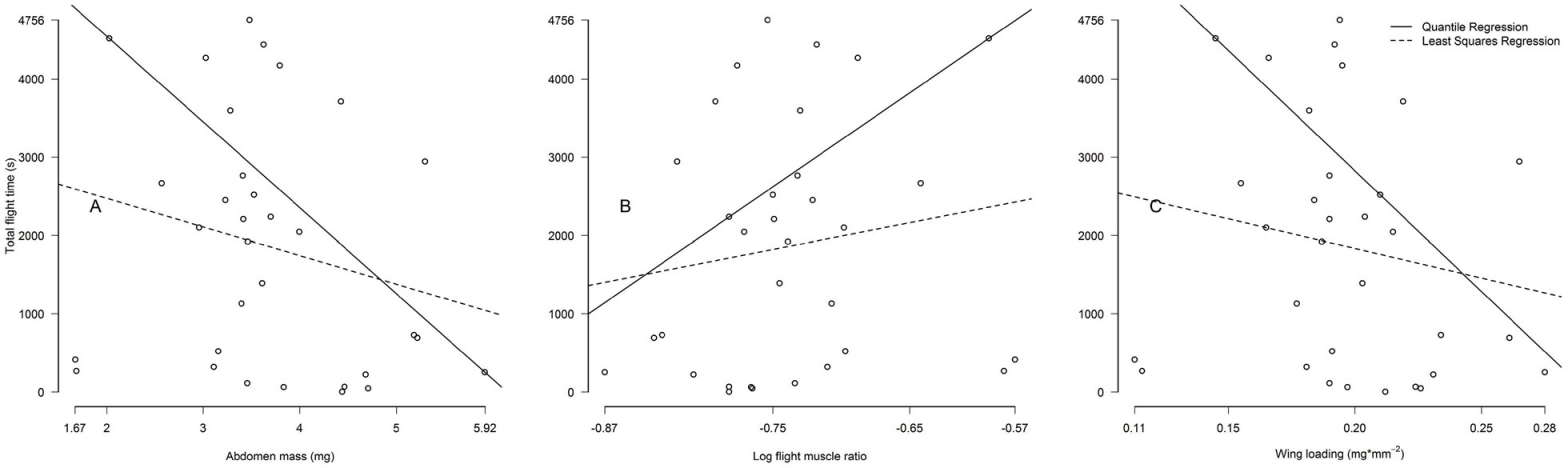
Flight duration and morphology. In claustral *S*. *invicta* queens, (A) heavier abdomens decrease a queen’s flight endurance due to (B) lower flight muscle ratios and (C) higher wing loading. Quantile regressions are through the top quartile.

Queens from different colonies did not differ in total flight duration (Kruskal-Wallis *p* = 0.45), log-transformed flight muscle ratio (ANOVA *p* = 0.18), abdomen mass (Kruskal-Wallis *p* = 0.09), or wing loading (Kruskal-Wallis *p* = 0.13), nor was colony identity a significant factor in any of the three quantile regressions (abdomen mass *p* = 0.40, flight muscle ratio *p* = 0.49, wing loading *p* = 0.76), and we therefore pooled the data. Among individual queens, the range of queen flight durations (P6) decreased with abdomen mass, so that the heaviest queens flew only for short time periods but light queens could have long or short flights (Fig 5A). When comparing maximum flight durations, the heaviest queens were able to fly only about 5% as long as the lightest (250 versus 4,900 seconds), with each milligram of abdominal loading decreasing maximum flight duration by about 18 minutes (quantile regression through upper quartile, Table 4A). Likewise, shorter maximum flight durations were associated with reduced flight muscle ratios (Fig 5B) and increased wing loading (Fig 5C), either of which, or both in combination, could be the mechanism driving shorter flight durations in heavier abdomens. Across all 32 queens, flight durations ranged over a hundredfold, from 47 seconds to over 79 minutes (ignoring one queen who flew for only four seconds). Notably, the longest flights lasted 30 to 75% longer than previous estimates of the maximum duration of *S*. *invicta* flights, which ranged from 45 minutes to 1 hour [52]. All measures of central tendency—least squares regressions and Spearman’s rank correlations—agreed in direction with the upper quartile results (Fig 5, Table 4B and 4C). As expected, however, they were not significant because minimum flight durations were the same for all flight morphologies. Any queen can fly for short periods, and only the range and maximum vary with a queen’s ability.

**Table 4:** Maximum flight duration versus queen morphology.

A. Quantile Regressions					
		Intercept	Slope	*p*	Corr. *p*
Abdomen mass		6742.350	-1096.915	0.024	0.054
Log flight muscle ratio		11633.347	12015.789	0.031	0.054
Wing loading		8988.231	-30815.385	0.018	0.054
B. Ordinary Least Squares Regressions					
	*r*^*2*^	Intercept	Slope	*p*	Corr. *p*
Abdomen mass	0.05	3202.1	-364.5	0.21	0.63
Log flight muscle ratio	0.02	4377	3405	0.41	0.68
Wing loading	0.03	3345	-7552	0.34	0.68
C. Spearman's Rank Correlations					
			*r*_*s*_	*p*	Corr. *p*
Abdomen mass			-0.27	0.14	0.42
Log flight muscle ratio			0.22	0.22	0.42
Wing loading			-0.26	0.16	0.42

All regressions were performed on *S*. *invicta* (*n* = 32).

Quantile regressions are through the upper quartile.

*Corr*. *p* shows *p* values corrected for experimentwise error.

### Tradeoff model

**Fig 6:**
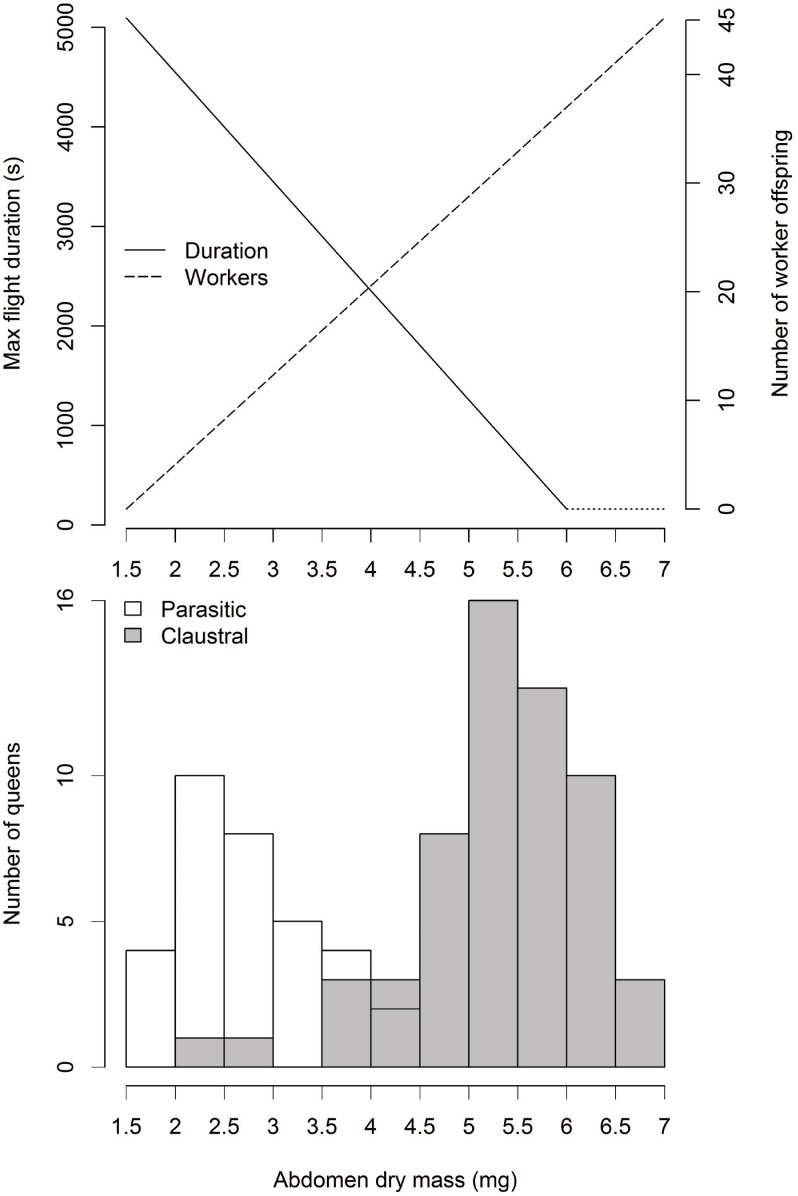
Fire ant reproduction-dispersal tradeoff model. Ant queens experience a tradeoff between flight ability and reproductive output mediated by abdomen mass. Claustral queens sacrifice flight endurance for colony founding ability, whereas parasitic queens cannot found colonies but can fly longer in search of hosts. Flight duration curve is adapted from Fig 5A, and durations above 6 mg abdomen mass are speculative. Worker production curve is adapted from the literature [68]. Abdomen mass histograms show *S*. *invicta* queens from the flight morphology comparisons.

Claustral and parasitic *S*. *invicta* queens appear to differ in their emphasis on flight versus reproduction (Fig 6). All else being equal, the heavier abdomens of claustral queens allow them to produce more workers in the early stages of colony founding, but the lighter abdomens of parasites should allow them to fly longer or farther in search of host colonies. The average claustral queen, with a 5.3 mg abdomen, would produce three times as many initial workers as the average parasitic queen with a 2.7 mg abdomen (31 versus 10 workers). At the same time, the parasitic queen should be able to fly over four times as long (3,800 versus 900 seconds) and 1.5 times as fast (0.9 versus 0.6 ms^-1^ on average), resulting in a 6-fold increase in flight range (3420 m versus 540 m). These predicted flight ranges agree with independent estimates that most claustral fire ant queens fly only a few hundred meters and for less than half an hour [34, 65].

## Discussion

Colonies of queen polymorphic ant species balance tradeoffs to allocate investment in different queen types. Parasitic queens are light and cheap to produce but have low reproductive outputs and cannot found colonies on their own. Claustral queens, on the other hand, are heavy and expensive but can produce many workers and found colonies independently. According to the Found or Fly hypothesis polymorphic queens should differ also in dispersal ability. Using morphological and experimental evidence, we document dispersal polymorphisms in two fire ant species, *Solenopsis geminata* and *S*. *invicta*. The heavier abdomens of claustral queens (Figs 2 and 3) cause them to have 32 to 38% lower flight muscle ratios, 55 to 63% higher wing loading, and 32 to 33% higher abdomen drag than conspecific parasites. If queen castes are developmentally determined, species can respond to this tradeoff by altering the morphology of the two queens. In *S*. *geminata*, for example, claustral queens develop 16% larger wings than parasitic queens (Fig 3D), offsetting some of the effects of heavier abdomens on wing loading. Heavy abdomens, through their effects on flight morphology, reduce maximum flight speed [52] and maximum flight duration (Fig 5). All else being equal, claustral queens should thus have reduced flight and dispersal ability relative to their parasitic counterparts (Fig 6). Our results suggest that dispersal tradeoffs may play a role in the evolution of alternative reproductive strategies.

Parasitic fire ant queens require specific nest sites—orphaned host colonies—that occur at low densities across the landscape (3 to 19 nests per hectare [27]). Claustral queens, in contrast, can potentially found a colony in any vacant patch of soil. If parasitic queens experience greater dispersal ability, it would grant them larger search areas and more search time to locate potential hosts. Assuming a purely horizontal flight and a constant maximum flight speed of 1.5 ms^-1^ [52], the 900 second flight of the average claustral queen would mean a potential colonization area of about 6 km^2^. If the same flight relationships hold in both queen types, the average parasitic queen could fly for 3,800 seconds and have a potential colonization area of 102 km^2^—a 17-fold difference. Using the lower and perhaps more realistic flight speeds of 0.9 and 0.6 ms^-1^ for parasitic and claustral queens, the difference more than doubles to 36-fold (36 versus 1 km^2^). Models of the reproductive success of the two queen types [27, 37], and the fitness return per investment for the colonies producing them, should therefore incorporate these search area differences.

Enhanced dispersal is not the only benefit associated with better flight ability. The leaner abdomens of parasitic queens should also result in greater maneuverability, which would likely increase their ability to evade predators [73–75] and to navigate aerial mating swarms and copulate [47, 76]. Their higher flight muscle ratios should also allow them to fly at lower temperatures than queens with heavier abdomens [44]. Indeed, in both fire ants the parasitic queens fly at cool times of the year, *S*. *geminata* in fall and *S*. *invicta* in late winter, while their claustral counterparts fly in spring and summer [27, 36].

The low weights of parasitic queens, and the apparent tradeoff between reproduction and dispersal, are probably not just an artifact of fall- and winter-reared queens being lighter due to reduced food availability. Small energy reserves are a common trait across parasitic ant species in general, regardless of when they fly [11, 26]. In *S*. *invicta*, for which we have detailed year-round census and metabolic data, fall is actually a time of abundance and colony growth, with colony energy input exceeding expenditures [34, 77]. In fact, colonies achieve their maximum annual size and nutrient stockpile in January, just before parasitic queens leave on mating flights [34, 77]. Fire ant colonies are therefore likely able to afford large and nutrient-rich parasitic queens were it profitable to do so, especially at the low numbers in which they are produced [78].

Quantifying dispersal ability is rarely straightforward. For example, claustral and parasitic queens may have qualitatively different flight behaviors and experience different flight environments. In *S*. *invicta* claustral queens fly up into the atmosphere and may take advantage of high altitude winds [65], while parasitic queens may disperse in low searching flights along the ground where wind speeds are reduced. Strict extrapolations based on flight performance in one queen type may thus not accurately describe flight in others. Further, laboratory flight experiments may not capture natural dispersal behavior in which queens may take off from varying heights by climbing vegetation, and fly several kilometers. In interpreting our flight duration experiment we analyzed the maximum performance range of queens (the upper quartile of flight durations) and further work is needed to explain flight variation below those maximum values. Finally, other factors besides flight may limit a queen’s ability to successfully disperse. In the most obvious example, parasitic queens can only disperse to areas where there are already populations of conspecifics to act as hosts, whereas claustral queens can colonize vacant habitats. More detailed field studies are needed to fully elucidate the costs and benefits of dispersal polymorphisms in these species.

Their diverse life histories and ability to generate multiple castes from the same genome make ants ideal organisms for studying morphological tradeoffs [20, 79]. By positing one such tradeoff involving flight morphology, the Found or Fly hypothesis provides a framework for addressing questions of ant dispersal and the evolution of alternative reproductive strategies. Although we focus on monogyne populations with only one queen per colony, similar tradeoffs may play out when comparing colonies with varying queen number [28]. Recognizing dispersal differences among all queen types, and knowing how colonies allocate investment among them, may allow us to better predict rates of range expansion in these invasive species. Investigations of male dispersal are likewise necessary for a complete understanding of gene flow and the evolution of alternative reproductive strategies in ants [80–82]. In the case of females, at least, it is clear that queen types represent not only different ways to found colonies, but also different ways to fly.

## Supporting Information

S1 Dataset Fire ant queen flight morphology and flight durations. (XLSX)
